# Lattice Model of Multilayer Adsorption of Particles with Orientation Dependent Interactions at Solid Surfaces

**DOI:** 10.3390/molecules26185622

**Published:** 2021-09-16

**Authors:** Andrzej Patrykiejew

**Affiliations:** Department of Theoretical Chemistry, Institute of Chemical Sciences, Faculty of Chemistry, MCS University, 20031 Lublin, Poland; andrzej.patrykiejew@poczta.umcs.lublin.pl

**Keywords:** multilayer adsorption, orientation-dependent interactions, Monte Carlo simulations

## Abstract

A simple lattice model has been used to study the formation of multilayer films by fluids with orientation-dependent interactions on solid surfaces. The particles, composed of two halves (A and B) were allowed to take on one of six different orientations. The interaction between a pair of differently oriented neighboring particles was assumed to depend on the degrees to which their A and B parts overlap. Here, we have assumed that the AA interaction was strongly attractive, the AB interaction was set to zero, while the BB interaction was varied between 0 and −1.0. The ground state properties of the model have been determined for the systems being in contact with non-selective and selective walls over the entire range of BB interaction energies between 0 and −1.0. It has been demonstrated that the structure of multilayer films depends on the strengths of surface potential felt by differently oriented particles and the interaction between the B halves of fluid particles. Finite temperature behavior has been studied by Monte Carlo simulation methods. It has been shown that the bulk phase phase diagram is qualitatively independent of the BB interaction energy, and has the swan neck shape, since the high stability of the dense ordered phase suppresses the possibility of the formation of disordered liquid-like phase. Only one class of non-uniform systems with the BB interaction set to zero has been considered. The results have been found to be consistent with the predictions stemming form the ground state considerations. In particular, we have found that a complete wetting occurs at any temperature, down to zero. Furthermore, the sequences of layering transitions, and the structure of multilayer films, have been found to be the same as observed in the ground state.

## 1. Introduction

The formation of multilayer adsorbed films on solid substrates and wetting phenomena have been studied for several decades, both experimentally, with different theoretical approaches, and computer simulation methods. A comprehensive description of the results can be found in many books and review articles [1,2,3,4,5,6,7,8,9]. Among the most frequently used theoretical models of adsorption are various lattice gas models [3,8,10,11,12,13,14,14]. Although such models are rather crude approximations to real systems, the emerging results resemble experimental results for multilayer adsorption quite well. The use of lattice models in studies of adsorption phenomena can be traced back to the beginning of the 20th century, when the Langmuir proposed his famous model of localized monolayer adsorption [15]. Later, Brunauer, Emmett and Teller [16] generalized the model of multilayer adsorption (BET model). A common feature of those early theoretical models was the negligence of attractive adsorbate–adsorbate interactions. Later, lattice models of multilayer adsorption involving attractive interactions between fluid particles were developed [10,13]. These models were applied to study adsorption of single component fluids and binary mixtures, using various mean-field theories [10,13,14,17,18,19], renormalization group methods [20,21] and computer simulations [11,13,14,22,23,24,25,26,26].

From the collected experimental and theoretical results it was established that the scenarios of the adsorbed film development are primarily controlled by the relative strength of adsorbate–adsorbate and adsorbate–solid interactions [12]. A comprehensive description of possible modes of the film growth was given already forty years ago by Pandit, Schick and Wortis [12]. Using the lattice gas model, these authors demonstrated that three different regimes of the film formation can be singled out. The first, called the strong substrate regime, corresponds to the systems in which the adsorbate–solid interaction dominates, and the film thickness diverges upon the approach to the bulk coexistence. At low temperatures, the adsorption occurs via an infinite number of first-order layering transitions. These transitions terminate in critical points, $T_{c}\left(n\right)$, where *n* is the layer number, and $T_{c}\left(n\right)$ converges to the roughening transition temperature, $T_{R}$, when n→∞. At higher temperatures the film thickness smoothly increases, and finally diverges, upon the approach to the bulk coexistence. The divergence of the film thickness implies a complete wetting to occur at any temperature down to zero. When the strength of the surface potential is lowered below a certain threshold value, the so-called intermediate substrate regime is entered. In this case, the wetting occurs only at finite temperatures, starting at the wetting temperature, $T_{w}$. Below $T_{w}$ the film thickness remains finite at the bulk coexistence, and it diverges only at, and above, $T_{w}$. Within the intermediate substrate regime, three different subregions can be identified, determined by the relative values of the roughening and the wetting temperatures. When $T_{w}<T_{R}$, the layering transitions are still present, but occur only at finite temperatures. When $T_{w}>T_{R}$, the system may exhibit the prewetting transition, followed by the first-order wetting transition at the bulk coexistence, or only the critical (continuous) wetting transition. Finally, when the surface potential becomes weak enough, the weak substrate regime is met, with incomplete wetting at any temperature.

Experimental and theoretical studies confirmed the predictions stemming from the presented above classification, and revealed several new features [9,12,23]. For example, it was shown that the layering transitions may involve a simultaneous condensation of more than one layer. However, the layers that condense together at low temperatures, may be separated by increasing entropy effects at higher temperatures, leading to the appearance of triple points [12,23]. In general, our understanding of multilayer films formation and wetting phenomena in systems with isotropic interactions is now quite advanced.

On the other hand, the effects of orientation dependent adsorbate–adsorbate interactions on the formation of multilayer films and wetting transitions have not been given much attention. A vast majority of experimental and theoretical studies has concentrated on adsorption of non-spherical molecules [27,28,29,30]. In such systems, the shape of adsorbate molecules gives rise to orientation-dependent interactions. Recently, experimental studies of the formation of multilayer structures by amphiphilic molecules have been reported [31,32,33]. However, these multilayer structures are not formed via adsorption processes, but rather in the bulk, due to the nature of interparticle interactions in such systems.

Another interesting class of systems with strongly orientational interactions consists of colloidal patchy particles [34,35,36] A special case of patchy particles, corresponds to Janus particles, in which there are only two regions of different chemical composition and physical properties [35]. Self-assembly of Janus particles has been studied experimentally [34,37,38] and with different theoretical tools [39,40], including computer simulations [41,42,43]. Adsorption of Janus particles at the fluid-fluid interface has been theoretically studied by several authors [44,45,46,47,48]. The only work dealing with adsorption of Janus particles at solid planar walls has been presented by Rosenthal and Klapp [39], who used the density functional theory. We shall return to their findings later in Section 5.

In this work, we consider a very simple lattice model of spherical Janus-like particles being in contact with solid substrates. Our main goal has been to elucidate the effects of orientation-dependent interactions on the structure of multilayer films, in systems characterized by different interaction energies between Janus particles and between Janus particles and the wall. In the case of a cubic lattice, each particle has been allowed to assume one of six orientations, and the interaction energy between a pair of neighboring particles has been assumed to depend on the degree to which the A and/or B halves overlap. In fact, the model is a generalization to three dimensions of the already considered two-dimensional model [49].

The paper is organized as follows. In the next section, we present the model used here. Then, in Section 3, we discuss its behavior in the ground state. In the following Section 4, we briefly describe the Monte Carlo method used to study bulk and non-uniform systems. The results of Monte Carlo simulations and their discussion is given in the Section 5. The paper concludes in Section 6, where we summarize our findings and present final remarks.

## 2. The Model

We consider a fluid placed on a regular lattice consisting of $DL^{2}$ sites arranged in *D* layers of $L^{2}$ sites each. The slab has been bounded on the top and the bottom by the same surfaces, being the source of the surface potential.

**Figure 1:**
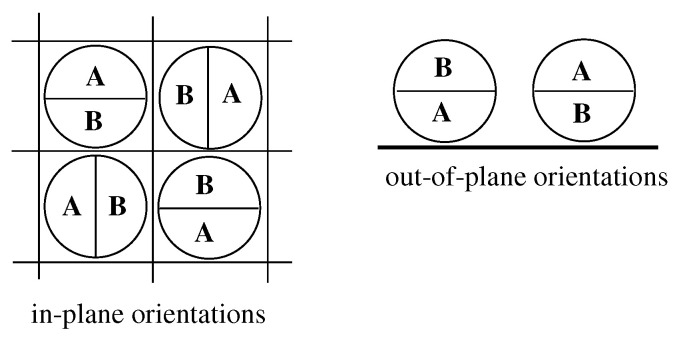
Six allowed orientations of particles. The left part shows the top view of the four in-plane orientations, while the right part shows the side view of the two out-of-plane orientations.

The fluid is assumed to consist of Janus-like particles with two halves, A and B. The interaction between a pair of particles is confined to the nearest neighbors only, and assumed to depend on the relative orientations of particles. Every particle can take on one of the six orientations, labeled by *k* (k=1,2,3…,6), as shown in Figure 1. Throughout this paper, and for the reason to be explained later, we call the orientations 1,2,3 and 4 as the in-plane orientations, while the orientations 5 and 6 as the out-of-plane orientations.

Let $u_{AA}$, $u_{AB}$ and $u_{BB}$ be the interaction energies corresponding to the relative orientations in which the AA, AB or BB halves face one another. Then, the interaction energy between a pair of particles located on neighboring sites can be written as

(1) $$\begin{array}{ccc} u(k_{i},k_{j},{\vec{r}}_{ij}) & = & w_{AA}\left(k_{i},k_{j},{\vec{r}}_{ij}\right)u_{AA}+w_{AB}\left(k_{i},k_{j},{\vec{r}}_{ij}\right)u_{AB}\\ & + & w_{BB}\left(k_{i},k_{j},{\vec{r}}_{ij}\right)u_{BB} \end{array}$$

In the above, $w_{AA}\left(k_{i},k_{j},{\vec{r}}_{ij}\right)$, $w_{AB}\left(k_{i},k_{j},{\vec{r}}_{ij}\right)$ and $w_{BB}\left(k_{i},k_{j},{\vec{r}}_{ij}\right)$ are the weights, determined by the degrees to which the AA, AB and BB regions overlap, and ${\vec{r}}_{ij}$ is the separation vector. In the case of a cubic lattice, there are six different separation vectors: ${\vec{r}}_{ij}=\left(\pm 1,0,0\right)$, (0,±1,0) and (0,0,±1).

Symmetry properties of the model imply that there are seven different values of $u(k_{i},k_{j},{\vec{r}}_{ij})$, summarized in Table 1.

The surface external potential $V_{k}\left(z\right)$, has been assumed to depend on the distance from the surface, *z*, and on the orientation of the particle with respect to the surface, *k*. Here, we assume that $V_{k}\left(z\right)$ has the following simple form:

(2) $$V\left(z,k\right)=\left\{\begin{array}{cc} \frac{V_{o,k}}{z^{3}} & z\leq 10\\ 0 & z>10 \end{array}\right.$$

with

(3) $$V_{o,k}=\left\{\begin{array}{cc} 0.5(V_{A}+V_{B}) & k=1,2,3,4\\ V_{A} & k=5\\ V_{B} & k=6 \end{array}\right.$$

with the above assumptions, the Hamiltonian of the model reads

(4) $$H=\sum_{\langle i,j\rangle }u\left(k_{i},k_{j},{\vec{r}}_{ij}\right)n_{i}n_{j}+\sum_{l=1}^{D}\sum_{i\in l}V\left(l,k_{i}\right)n_{i}-\mu \sum_{i=1}^{DL^{2}}n_{i}$$

In the above, $n_{i}=1\left(0\right)$, when the *i*-th site is occupied (empty), the first sum runs over all pairs of nearest neighbors, V(l,k) is just V(z,k) with the distance from the surface expressed in lattice spacings, and μ is the chemical potential.

Now, for the fixed *D* and *L*, the free energy of the system is given by

(5) $$F_{L,D}\left(T,\mu \right)=-kT\ln Tr\exp\left(-\beta H\right)$$

with β=1/kT.

In principle, we are interested in the behavior of the model in the thermodynamic limit, when D,L→∞. Under such conditions, the bulk and the surface excess free energies (per site) can be expressed as [12,23]

(6) $$f_{b}\left(T,\mu \right)=\lim_{D,L\to \infty }\frac{F_{L,D}\left(T,\mu \right)}{DL^{2}}$$

and

(7) $$f_{ex}\left(T,\mu \right)=\lim_{D,L\to \infty }\frac{F_{L,D}\left(T,\mu \right)-DL^{2}f_{b}\left(T,\mu \right)}{2L^{2}}$$

The factor 2 in the denominator of the last equation results from the assumed presence of two surfaces, at the bottom and at the top of the system.

Here we confine the discussion to the systems with $u_{AA}=-1.0$, $u_{AB}=0$, and with different $u_{BB}\in \left[-1.0,0\right]$. Thus, all inter-reactions are non-repulsive, but, in general, the BB attraction has been assumed to weaker than the AA attraction. This choice of parameters seems quite well suited to represent Janus-like particles [50,51,52].

In order to show how the adsorption behavior is affected by the properties of the surface potential, we have considered systems with $V_{A}=V_{B}$, $V_{A}>V_{B}$ and $V_{A}<V_{B}$.

## 3. Ground State Properties

The bulk free energy $f_{b}\left(T,\mu \right)$ can be written as

(8) $$f_{b}\left(T,\mu \right)=u_{b}-Ts_{b}-\mu \rho_{b}$$

with $u_{b}$, $s_{b}$ and $\rho_{b}$ being the bulk potential energy, entropy and density, respectively. Similarly, the surface excess free energy and $f_{ex}\left(T,\mu \right)$ is given by

(9) $$f_{ex}\left(T,\mu \right)=u_{ex}-Ts_{ex}-\mu \rho_{ex}$$

where $u_{ex}$, $s_{ex}$ and $\rho_{ex}$ denote the surface excesses of potential energy, entropy and density, respectively.

**Figure 2:**
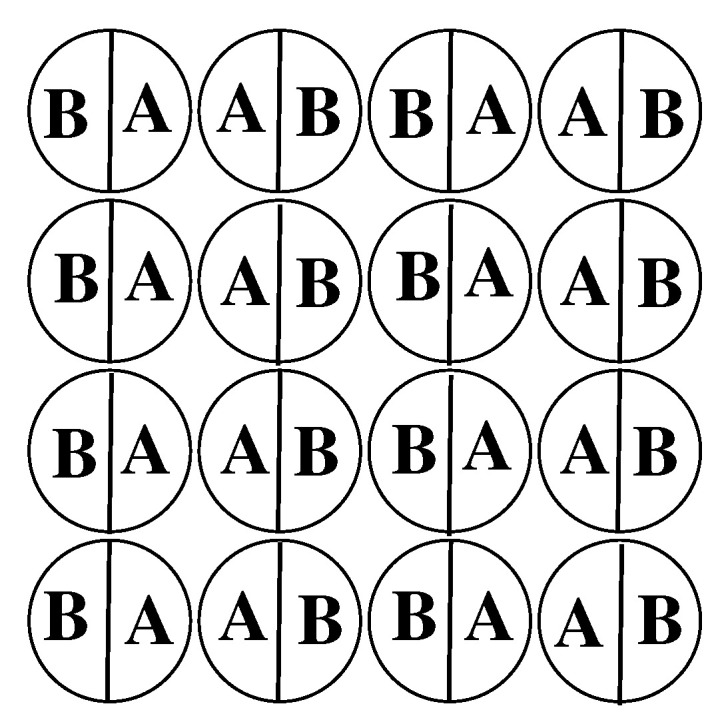
The structure of the stripped phase with two in-plane orientations of particles.

In the ground state, the free energy lacks the entropic term, since T=0. In particular, the bulk free energy is given by

(10) $$f_{b}\left(0,\mu \right)=\left\{\begin{array}{cc} 0, & \mu \leq \mu_{o}\\ \mu_{o}-\mu , & \mu >\mu_{o} \end{array}\right.\;\;,$$

with $\mu_{o}$ being the chemical potential at the coexistence between the dilute and condensed phases at T=0. Of course, $\mu_{o}$ depends on the structure of the condensed phase. In the systems considered here, the condensed phase is well ordered, and built of layers consisting of the same sequence of rows of particles, with alternate orientations …AB-BA-AB-BA… (cf. Figure 2).

The energy (per site) of this ordered structure at T=0 is equal to

(11) $$u_{b}=1.5\left(u_{BB}+u_{AA}\right),$$

and the bulk coexistence is located at the chemical potential $\mu_{o}=u_{b}$.

Note that in the case of $u_{BB}=-1.0$, $\mu_{o}=-3.0$, just like in the system with isotropic interactions [23], since AB contacts do not appear at all, while the AA and BB pairs interact with the same energy.

In the ground state, the adsorbed film at the specified chemical potential is expected to consist of a certain number of occupied layers, with the surface excess free energy (per site) given by

(12) $$f_{ex}\left(0,\mu^{\prime }\right)=\min_{l}\min_{{\left\{k\right\}}_{l}}\left[u_{ex}^{l}\left({\left\{k\right\}}_{l}\right)-\mu^{\prime }l\right]$$

In the above $u_{ex}^{l}\left({\left\{k\right\}}_{l}\right)$ is the surface excess energy (per site) that depends on the particle orientations in all occupied layers, and $\mu^{\prime }=\mu -\mu_{o}$. Of course, as long as we consider adsorption from a dilute (gas-like) phase, we need to take into account only the values of $\mu^{\prime }$ lower than zero.

The lowest energy of interparticle interactions in an individual layer is equal to $u_{BB}-1$, and is reached for the configurations in which all particles assume the same out-of-plane orientation with their A or B halves down (k=5 or 6), or when the layer consists of stripes, as those shown in Figure 2, with two in-plane orientations, k=1 and k=3 or k=2 and k=4.

In order to determine the energy of the film consisting of *n* filled layers it is necessary to include the contributions due to the fluid-substrate interaction and the interactions between the particles in adjacent layers. Thus, the energy of the film can be written as

(13) $$u_{n}\left({\left\{k\right\}}_{n}\right)=\sum_{l=1}^{n}V\left(l,k_{l}\right)+n\left(u_{BB}-1.0\right)+\sum_{l=2}^{n}u_{inter}\left(k_{l-1},k_{l}\right)$$

where $u_{inter}\left(k_{l-1},k_{l}\right)$ is the contribution to the film energy due to interaction between the particles in layers l−1 and *l*. In the considered here case of $u_{AB}=0$, $u_{inter}\left(k_{l-1},k_{l}\right)$ takes on one of the values given in Table 2.

In Table 2, we have marked the configurations with in-plane orientations of particles as 1, since the interlayer energy does not depend on the orientations of stripes.

One should note that for any $u_{BB}>-1$, the stacking 65 in neighboring layers is energetically most favorable. However, the actual stacking of orientations in multilayer films depends on the particular values of $V_{A}$, $V_{B}$ and $u_{BB}$. An important parameter is $\Delta V=V_{A}-V_{B}$. The case of ΔV=0 corresponds to non-selective walls, while the cases with ΔV>0 and ΔV<0 correspond to selective walls.

At first we discuss the systems with ΔV=0, with $V_{A}=V_{B}=V_{o}$. In general, there are three structures of the film with *n* occupied layers possible. In the first, the particles in each layer have in-plane orientations, and the film energy is equal to

(14) $$u_{in}\left(n\right)=\left(1.5n-0.5\right)\left(u_{BB}-1.0\right)+V_{o}\sum_{i=1}^{n}i^{-3}$$

When the particles in the film assume out-of-plane orientations, the film energy for even and odd number of occupied layers is given by different expressions. As long as $u_{BB}>-1.0$, the stacking that minimizes the energy is 6565…, due to strong interlayer attraction. Only in the case of $u_{BB}=-1.0$ the stacking 5656… as well as the stacking with in-plane orientations in all layers give the same film energy.

The potential energy of the film with an even number of layers is given by

(15) $$u_{out,even}\left(n\right)=1.5n\left(u_{BB}-1.0\right)-u_{BB}+V_{o}\sum_{i=1}^{n}i^{-3}\;,$$

while for the odd number of layers, the film energy is given by Equation (14).

Having the film energies we can readily derive the expressions which give the chemical potential values at the transition points between the states of different numbers of occupied layers. In general, the following different layering transitions are possible:

(16) 0→n with odd n,μ1′(0→n)=−0.5(uBB−1)n+Von∑i=1ni−3

(17) 0→n with even n,μ2′(0→n)=−uBBn+Von∑i=1ni−3

(18) n→n+1 with odd n,μn→n+1′=−0.5(uBB+1)+Vo(n+1)−3

(19) n→n+1 with even n,μn→n+1′=0.5(uBB+1)+Vo(n+1)−3

and

(20) n→n+2 with even n,μn→n+2′=Vo2[(n+1)−3+(n+2)−3]

In order to determine the actual sequence of transitions in a given system one needs to compare the excess free energies of the states with different numbers of occupied layers, since only the states that minimize the system free energy are stable.

Before we present the examples of ground state phase diagrams, let us note that the 0→1 transition is possible only for $V_{o}<-1/0.875\approx -1.142857143$, independently of $u_{BB}$. However, the chemical potential at which this transition is located depends on the both $V_{o}$ and $u_{BB}$. For any $V_{o}>-1/0.875$, the first layering transition involves a simultaneous condensation of two (or more) layers. In general, similarly to the systems systems with isotropic interactions [12], the number of simultaneously condensing layers increases when $u_{BB}$ becomes lower, i.e., when the BB attraction becomes stronger. An ultimate limit of 0→n transitions corresponds to n=∞, and it determines the region of complete wetting at T=0. Assuming an infinite range of the surface potential, this limit corresponds to the following relation between $u_{BB}$ and $V_{o}$

(21) $$u_{BB}/V_{o}=\zeta \left(3\right)\approx 1.20206$$

where ζ(3) is the Riemann function [53]. Whenever $u_{BB}/V_{o}$ is lower than ζ(3), the system exhibits a complete wetting at T=0. Thus, in the particular case of $u_{BB}=0$, a complete wetting should occur even for very weakly attractive surfaces.

**Figure 3:**
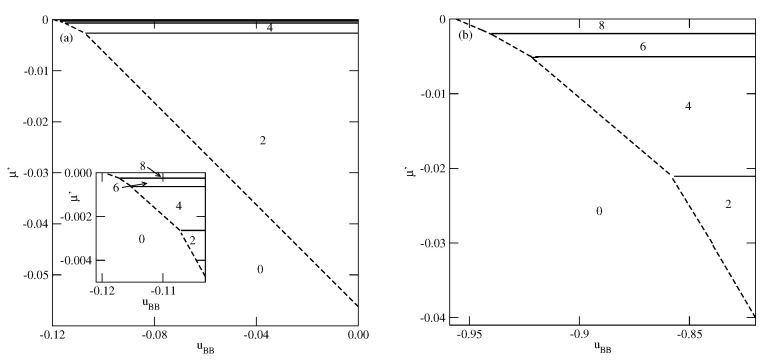
The ground state phase diagrams for the systems with $V_{o}=-0.1$ (part (**a**)) and −0.8 (part (**b**)). The numbers in the figure mark the regions of different film thickness. The inset to part a shows the magnified upper left corner of the phase diagram. In part (**b**), only the region corresponding to $u_{BB}<-0.82$ is shown.

**Figure 4:**
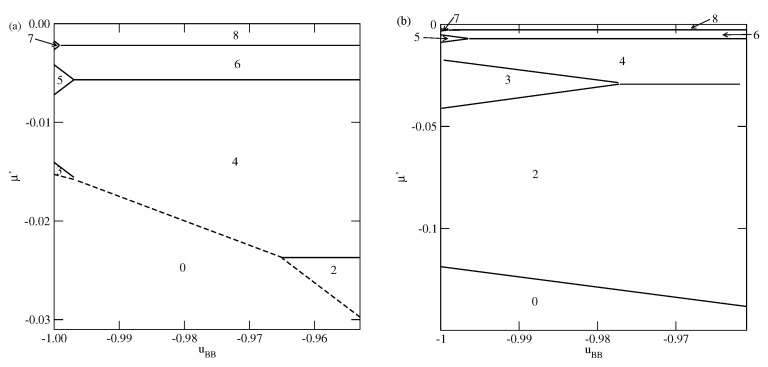
The ground state phase diagrams for the systems with $V_{o}=-0.9$ (**a** panel) and −1.0 (**b** panel). The numbers mark regions of different film thickness. Only the regions with $u_{BB}$ close to −1.0 are shown.

**Figure 5:**
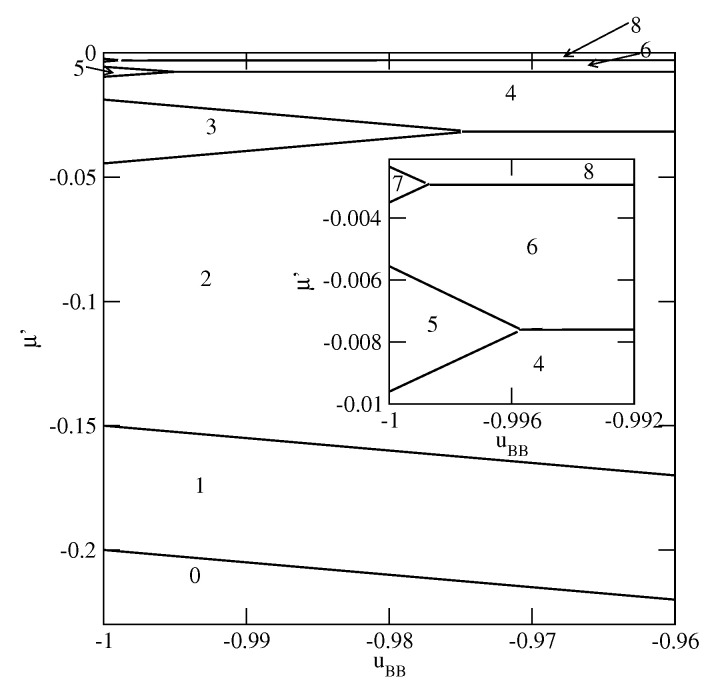
The ground state phase diagrams for the systems with $V_{o}=-1.2$. The inset shows the behavior for $u_{BB}$ close to −1.0.

Figure 3, Figure 4 and Figure 5 present the examples of ground state phase diagrams, obtained for different values of $V_{o}$, and over the entire range of $u_{BB}$ between 0 and −1. The phase diagrams for $V_{o}=-0.1$ (Figure 3a) and −0.8 (Figure 3b) are qualitatively the same, and demonstrate that independently of $u_{BB}$, the film grows via a sequence of layering transitions leading to the films of only even numbers of occupied layers. These phase diagrams also demonstrate that the range of $u_{BB}$ allowing for the formation of wetting layers at T=0 increases when the surface potential becomes stronger. In the case of $V_{o}=-0.1$, it is confined to the values of $u_{BB}$ not lower than about −0.11, while in the system with $V_{o}=-0.8$, a complete wetting occurs over much wider range of $u_{BB}$, between zero and about −0.96. One should note that the reported here values of $u_{BB}$, which delimits the regions of complete and incomplete wetting, are slightly different than predicted by Equation (21), due to the assumed finite range of the surface potential. The results demonstrate also that the first layering transition, 0→n, involves a simultaneous condensation of increasing number of layers when $u_{BB}$ becomes lower.

The phase behavior changes when the strength of the fluid–wall potential becomes high enough to ensure a complete wetting over the entire range of $u_{BB}$, between 0 and −1.0. In Figure 4, we present two ground state phase diagrams, for $V_{o}=-0.9$ (Figure 4a) and $V_{o}=-1.0$ (Figure 4b).

In the systems with $V_{o}=-0.9$, and $u_{BB}>-0.965$, the first layering transition leads to the formation of a bilayer, while for lower $u_{BB}$, down to about −0.9966, a simultaneous condensation of four layers occurs. However, for still lower $u_{BB}$, the first layering transition involves a simultaneous condensation of only three layers. When $u_{BB}$ approaches −1.0, the formation of thicker films occur via an increasing number of n→n+1 transitions. We recall that in the particular case of $u_{BB}=-1.0$, the film potential energy has the same value as the system with isotropic interactions, in which the film develops in a layer-by-layer mode [12,23]. When $V_{o}=-1.0$ (Figure 4b), the first layering transition leads to the bilayer film, for any $u_{BB}$ between 0 and −1.0. It results from the increased strength of the surface potential. The same was found in the isotropic system with $V_{o}=-1.0$ [23]. Again for $u_{BB}$ approaching −1.0, thick films develop via a sequence of n→n+1 transitions.

As soon as $V_{o}$ becomes lower than −1/0.875, the first layering transition leads to the formation of a monolayer film, followed by the 1→2 transition, for any $u_{BB}\in \left[-1.0,0\right]$ (Figure 5). However, the sequence of layering transition leading to the formation of thicker films, depends on $u_{BB}$. Similarly as in the systems with $V_{o}>1/0.875$, the film with odd numbers of layers appears only when $u_{BB}$ is sufficiently close to −1.0. For example, when $V_{o}=-1.2$, the formation of films with odd numbers of filled layers is possible only when $u_{BB}$ is lower than about −0.97. In the case of much stronger surface potential, with $V_{o}=-3.0$, this region is only slightly wider and requires $u_{BB}$ to be lower than about −0.94. However, for any $u_{BB}>-1.0$, the sequence of layering transitions n→n+1 terminates at finite $n_{max}$, and a further film growth occurs via n→n+2 transitions. Only when $u_{BB}=-1.0$ the film thickness grows in a layer-by-layer mode.

In the case of selective surfaces with ΔV>0, the first layer attains the lowest energy

(22) $$u_{1}\left(6\right)=V_{B}\left(1\right)+u_{BB}-1.0\;,$$

when all particles have their B halves oriented towards the surface (k=6).

In the bilayer film, the 65 stacking of the energy equal to

(23) $$u_{2}\left(6,5\right)=V_{B}\left(1\right)V_{A}\left(2\right)+2\left(u_{BB}-1.0\right)-1.0$$

is stable as long as $\Delta V=V_{A}-V_{B}<8$. Only for ΔV>8, the stacking 66 of the energy

(24) $$u_{2}\left(66\right)=V_{B}\left(1\right)+V_{B}\left(2\right)+2\left(u_{BB}-1.0\right)\;,$$

becomes stable. The stackings 55 and 56 can not appear at all.

The stable monolayer of the stacking 65 appears when $V_{B}-0.125V_{a}<-1.0$.

**Figure 6:**
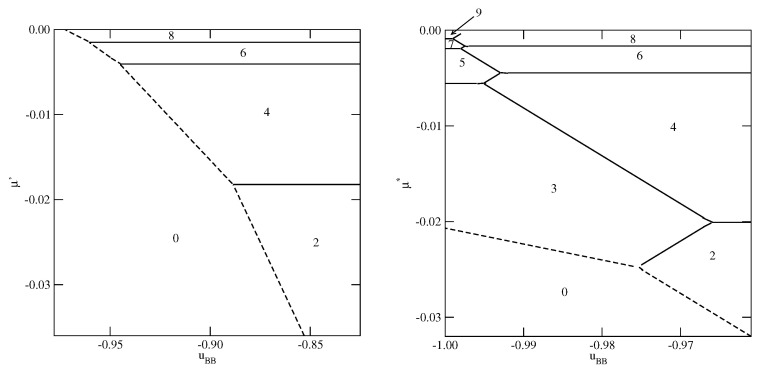
The ground state phase diagrams for the systems with $V_{A}=-0.2$ and different values of $V_{B}=-0.9$ (**left** panel) and −1.0 (**right** panel). The numbers mark the numbers of layers stable in different regions.

**Figure 7:**
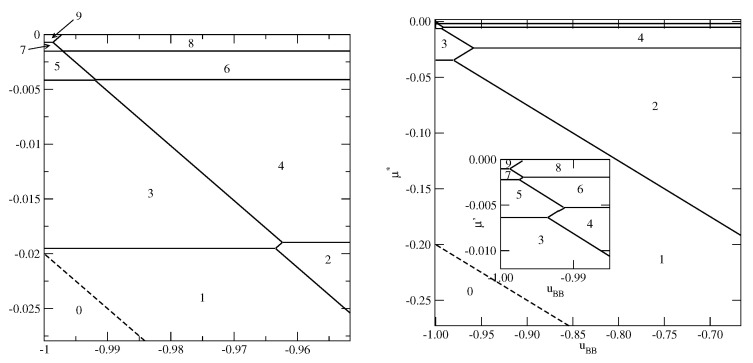
The ground state phase diagrams for the systems with $V_{A}=-0.01$ and $V_{B}=-1.02$ (**left** panel) and with $V_{A}=-0.2$ and $V_{B}=-1.2$ (**right** panel). The inset in the right panel shows the behavior in the region of $u_{BB}$ close to −1.0. The numbers mark in the figure the numbers of occupied layers in different regions.

**Figure 8:**
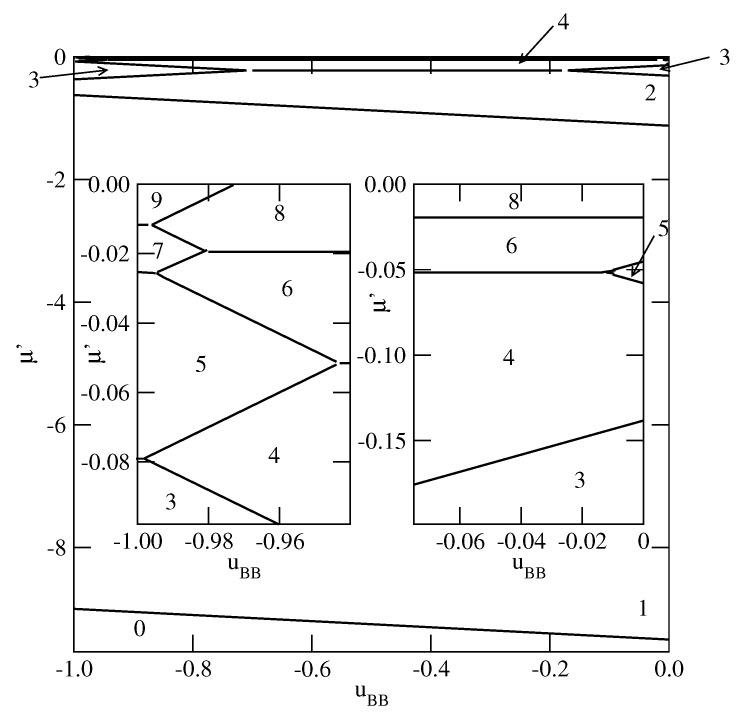
The ground state phase diagram for the system with $V_{A}=-5.0$ and $V_{B}=-10.0$. The right and left insets show the parts of the phase diagram corresponding to $u_{BB}$ close to 0 and to −1.0, respectively. The numbers mark the numbers of layers stable in different regions.

The examples of ground phase diagrams for selected systems with ΔV>0 are presented in Figure 6, Figure 7 and Figure 8. Figure 6 presents the phase diagrams for two systems, with $V_{A}=-0.2$ and $V_{B}=-0.9$ (left panel) and −1.0 (right panel), in which monolayer films do not appear.

The systems with $V_{B}=-0.9$ exhibit a complete wetting only when $u_{BB}$ greater than about −0.974 and shows the presence of only even numbers of occupied layers, with the stacking (6565…). On the other hand, when $V_{B}=-1.0$, the system exhibits a complete wetting for any $u_{BB}$, down to −1.0, and shows the formation of films with only even numbers of layers for $u_{BB}>-0.975$. When the BB attraction becomes stronger ($u_{BB}<-0.975$) the films with odd numbers of layers are also present. The first layering transition involves a simultaneous condensation of three layers. For $u_{BB}$ sufficiently close to −1.0 only odd numbers of layers appear, and the stacking remains the same (6565…). This behavior is different from that observed in the systems with $V_{A}=V_{B}$, for which the film was found to grow in a layer-by-layer mode only when $u_{BB}\to -1.0$.

Figure 7 presents two examples of phase diagrams demonstrating that when $V_{B}\left(1\right)-V_{A}\left(2\right)$ becomes even slightly lower than −1.0, or when $V_{B}$ becomes lower than −1/0.875 a monolayer film is stable over the entire range of $u_{BB}$.

By changing the strengths of the surface potential felt by the A and B halves, different scenarios of the film development can be observed. As an example, we show in Figure 8 the phase diagram for the system with $V_{A}=-5.0$ and $V_{B}=-10.0$.

The behavior in the region of $u_{BB}$ close to −1.0 is quite the same as in other systems with sufficiently large ΔV. On the other hand, for $u_{BB}$ close to zero a certain number of n→n+1 transitions occurs. Only when the film becomes sufficiently thick, it develops via the n→n+2 transitions, with even values of *n*.

**Figure 9:**
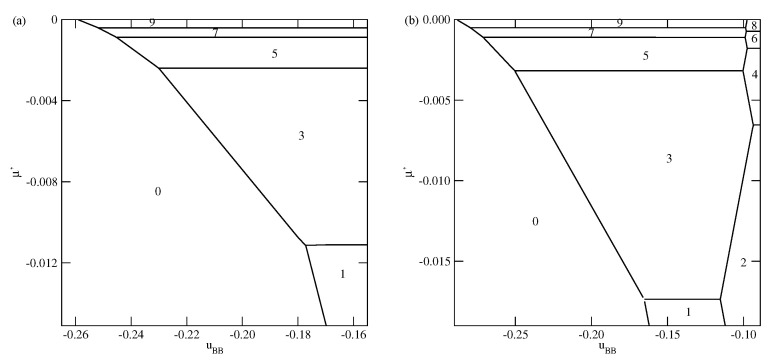
The ground state phase diagrams for the systems with $V_{A}=-0.6$ and $V_{B}=0.0$ (**a** panel) and with $V_{A}=-0.6$ and $V_{B}=-0.1$ (**b** panel). The numbers mark the numbers of layers stable in different regions.

**Figure 10:**
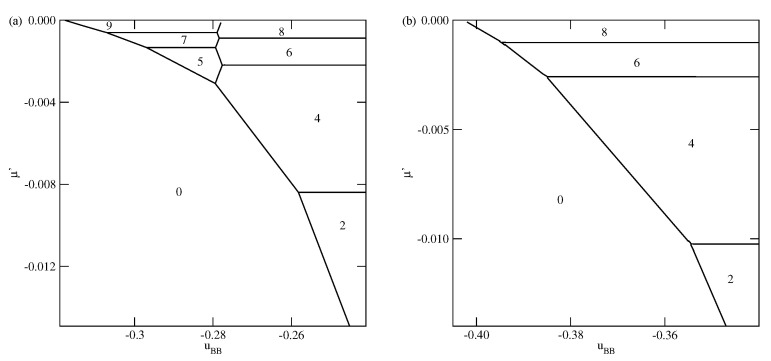
The ground state phase diagrams for the systems with $V_{A}=-0.6$ and $V_{B}=-0.2$ (**a** panel) and with $V_{A}=-0.6$ and $V_{B}=-0.3$ (**b** panel). The numbers mark the numbers of layers stable in different regions.

Finally we present the results for selective surfaces with ΔV<0. The most important difference is that for ΔV<0 the formation of stable monolayer films may occur for considerably weaker surface fields. Since the monolayer reaches the lowest energy when the particles assume the orientation 5, while the bilayer has the lowest energy for the stacking 65, the stable monolayer appears whenever $\Delta V+0.875V_{A}<-1$. In Figure 9 and Figure 10, we present a series of ground state phase diagrams obtained for the systems with $V_{A}=-0.6$ and different $V_{B}$, between 0.0 and −0.3.

When $V_{B}=0.0$ only odd numbers of occupied layers are stable, and the complete wetting at T=0 occurs only for $u_{BB}$ greater than about −0.26. Independently of the film thickness, all particles in the first layer assume the same orientation with k=5, i.e., with their A halves pointing towards the surface. Each of the subsequent layering transition adds two layers with the orientations 6 and 5, due to strong interlayer attraction. Since the assumed here strengths of the fluid–wall interactions are rather small, a gradual decrease of $u_{BB}$ causes the first layering transition to involve a simultaneous condensation of an increasing number of layers. When $V_{B}=-0.1$, the ground state behavior becomes different (see Figure 9b). Namely, the films with even numbers of occupied layers appear only for sufficiently weak attractive BB interaction. It should be noted that the 0→1 transition is preserved, and the formation of a bilayer involves the reorientation of particles in the first layer from k=5 to k=6. In the second layer all particles assume the orientation with k=5, since this stacking is energetically favorable due to strong interlayer attraction. When the BB interaction becomes strong enough, multilayer films with only odd numbers of filled layers appear. Over a very narrow range of $u_{BB}$, between the regimes with even and odd numbers of occupied layers, the films grow in a layer-by-layer mode.

In the case of $V_{B}=-0.2$, the phase behavior changes, since the 0→1 transition does not appear at all. Instead, a series of 0→n transitions, with even and odd *n*, occurs (see Figure 10a). The formation of thicker films occurs in a similar way as in the systems with $V_{B}=-0.1$, but the crossover between the regimes in which even and odd numbers of layers are stable is shifted towards considerably lower $u_{BB}$, close to −0.28.

In the system with $V_{B}=-0.3$ only the films with even numbers of occupied layers appear (see Figure 10b).

The observed in Figure 9 and Figure 10 changes in the ground state behavior, can be easily understood by taking into account a competition between the contributions to the film energy resulting from the fluid–wall and interlayer interactions. When $V_{B}$ becomes closer to $V_{A}$, the interlayer attraction dominates, but its role becomes lower when the BB attraction increases.

**Figure 11:**
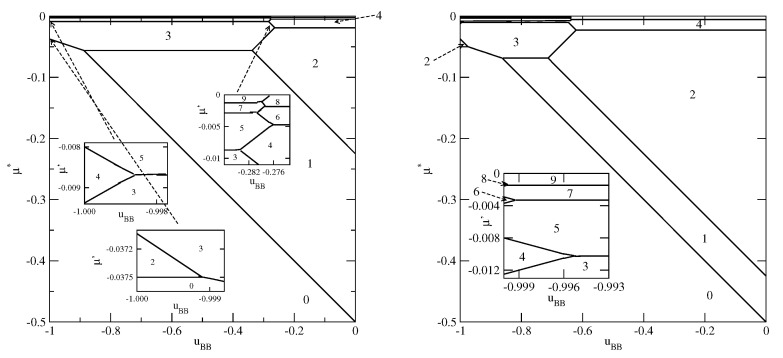
The ground state phase diagrams for the systems with $V_{A}=-1.0$ and $V_{B}=-0.6$ (**left** panel) and with $V_{A}=-1.0$ and $V_{B}=-0.8$ (**right** panel). The numbers mark the numbers of layers stable in different regions.

When $V_{A}$ becomes strong enough to ensure a compete wetting over the entire range of $u_{BB}$, different scenarios of the film development appear. We present explicit results for the systems with $V_{A}=-1.0$ and different values of $V_{B}$. For $V_{B}$ between 0.0 and about −0.43, the films consist of odd numbers of layers only, while for $V_{B}$ between about −0.43 and about −0.59, the behavior is qualitatively the same as shown in Figure 7. As soon as $V_{B}$ becomes equal to −0.6, or assume still lower values, the layer-by-layer growth occurs, but only for $u_{BB}$ very close to −1.0 (see Figure 11). This is illustrated by the insets to Figure 11. It is evident that in the systems with $V_{B}=-0.8$, the layer-by-layer growth occurs over a wider region of $u_{BB}$ than in the systems with $V_{B}=-0.6$. When $V_{B}$ becomes closer to $V_{A}$, the ground state behavior gradually approaches that found for non-selective walls.

## 4. Monte Carlo Simulations

We have used a standard Monte Carlo simulation method in the grand canonical ensemble [54,55], to study the bulk behavior of the model and the formation of multilayer films.

In the case of bulk systems, the cubic simulation cell of the size with L=D=20, and with periodic boundary conditions applied in all three directions, has been used. It has been found that the chosen system size was sufficient to estimate the phase diagrams, by measuring the isotherms at different temperatures. In some cases, we have used larger systems with L=D=30 and 40. The quantities recorded, included the system density

(25) $$\rho_{b}=\frac{1}{L^{3}}\langle \sum_{i=1}^{|L^{3}}n_{i}\rangle \;,$$

the densities of differently oriented particles

(26) $$\rho_{b,k}=\frac{1}{L^{3}}\langle \sum_{i=1}^{|L^{3}}n_{i}\delta \left(k_{i}-k\right)\rangle \;,$$

the average potential energy per site

(27) $$\langle u\rangle =\frac{1}{L^{3}}\langle \sum_{ij}n_{i}n_{j}u\left(k_{i},k_{j},{\vec{r}}_{ij}\right)\rangle \;,$$

the heat capacity

(28) $$C_{V}=\frac{1}{T^{2}}\left[\langle H^{2}\rangle -{\langle H\rangle }^{2}\right]\;,$$

and the density susceptibility

(29) $$\chi_{\rho }=\frac{1}{T}\left[\langle \rho^{2}\rangle -{\langle \rho \rangle }^{2}\right]\;.$$

In order to obtain reliable results at any state point, determined by the temperature and the chemical potential, the system had to be well equilibrated. This has been achieved by performing runs using $10^{6}$–$10^{7}$ Monte Carlo steps. Each Monte Carlo step involved 10 L${}^{3}$ attempts to either create a particle of randomly chosen orientation in a randomly chosen position, or to annihilate one of also randomly chosen particles.

In order to determine the behavior of closed packed bulk systems, we have used Monte Carlo method in the canonical ensemble. In this case, the only possible move involved the change of orientation of randomly chosen particle.

On the other hand, in the study of adsorption phenomena the system was a slab consisting of $DL^{2}$ sites. The width of the slab has been chosen as equal to D=60. With the assumed cut-off distance of the surface potential, $z_{cut}=10$, the system interior with *z* between 15 and 45, was not affected by the surface potential and could be considered as having the properties of the bulk. The linear dimension of each layer was set to L=40, and we have applied periodic boundary conditions to each layer. The basic recorded quantities were the surface excess density

(30) $$\rho_{ex}=\frac{1}{2}\sum_{l=1}^{D}\left(\rho \left(l\right)-\rho_{b}\right)$$

and the surface excesses of differently oriented particles

(31) $$\rho_{ex}\left(k\right)=\frac{1}{2}\sum_{l=1}^{D}\left(\rho_{k}\left(l\right)-\rho_{b,l}\right)$$

In the above, ρ(l) and $\rho_{k}\left(l\right)$, are the total layer density and the layer density of particles with the *k*-th orientation. The values of $\rho_{b}$ and $\rho_{k}\left(l\right)$ were obtained by averaging the corresponding layer densities for *l* between 15 and 45. We have also recorded the total density profiles, ρ(l), and the density profiles of differently oriented particles $\rho_{k}\left(l\right)$.

Since the strongest fluctuations occur in the layers rather close to the surface, we have used the preferential sampling of the surface region. Namely, the first 10 layers adjacent to the surfaces, at the bottom and at the top of the simulation cell, have been sampled 10 times more often than the system interior, consisting of the layers between 15 and 45. The layers 11–14 and 46–49, have been sampled 5 times more frequently than the system interior. One should note that fluctuations deep in the bulk are considerably smaller than in the surface region, in particular, at low temperatures. Significant fluctuations in the bulk occur at the temperatures close to the critical point. However, the considered here bulk systems do not exhibit critical points, as it is shown below in Section 5.1.

## 5. Finite Temperature Behavior

### 5.1. Bulk Systems

**Figure 12:**
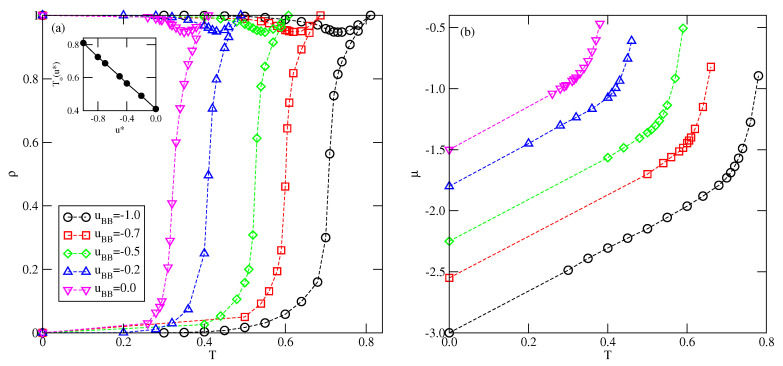
The T−ρ (**a**) and T−μ (**b**) projections of the Bulk phase diagrams for different values of $u_{BB}$, given in the part (**a**).

**Figure 13:**
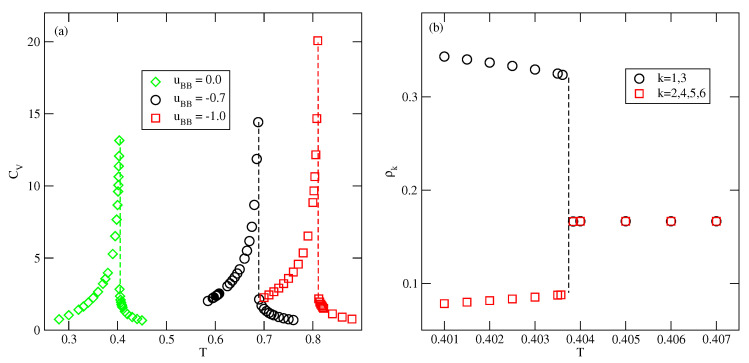
(Part (**a**)) The examples of heat capacity curves for the closed packed systems with different $u_{BB}$, obtained from the canonical ensemble Monte Carlo simulations. (Part (**b**)) Temperature changes of the densities $\rho_{k}$ for the system with $u_{BB}=0$.

In the case of lattice gas model with isotropic nearest neighbor interactions, the bulk phase exhibits a gas–(lattice) liquid transition at low temperatures, which terminates in the critical point. Moreover, the chemical potential at the bulk coexistence is temperature independent [12]. The situation is quite different in the present model involving orientation dependent interactions. Figure 12 presents the phase diagrams obtained for the bulk systems characterized by different values of $u_{BB}$, and shows that the phase behavior is qualitatively the same for any $u_{BB}$ between −1.0 and 0.0. These systems undergo the first-order transition between the disordered fluid phase and the orientationally ordered condensed phase of high density. In the systems considered here, the structures in which two A halves face each other are favored, and this effect is enhanced when the attractive BB interaction becomes stronger. The structure of a perfectly ordered condensed phase of density ρ=1.0 has been shown in Figure 2. A high stability of the ordered phase caused that phase diagrams for the systems considered here do not show the presence of critical point. The first-order transition between the dilute and condensed phases terminates in the point at which the system with the density equal to unity (fully filled lattice) exhibits the order-disorder transition, at $T_{o}(u_{BB}$, between the orientationally ordered and orientationally disordered phases. The phase diagrams of all systems assume the swan-neck shape. Figure 12 shows that when $u_{BB}$ decreases from zero to −1.0, the condensed phase retains stability over increasing ranges of temperatures, and $T_{o}\left(u_{BB}\right)$ also increases when $u_{BB}$ changes between 0.0 and −1.0. The transition at $T_{o}\left(u_{BB}\right)$ has been found to be of the first order. This has been confirmed by the calculations of temperature changes of the heat capacity and the densities of differently oriented particles. In Figure 13a, we shows heat capacity curves for three systems with $u^{*}=0.0$, −0.7 and −1.0. In all cases, the heat capacity exhibits finite discontinuous jumps at the transition temperature.

Figure 13b shows the temperature changes of the densities of differently oriented particles recorded for the system with $u_{BB}=0$. The results confirm that the transition is discontinuous, and show that in the low temperature ordered phase the two orientations (1 and 3) are dominating, while at the temperatures above the order-disorder transition all six orientations appear with the same probability.

### 5.2. Non-Uniform Systems

The lack of usual gas-liquid transitions has a great effect on the formation of thick films and the wetting behavior. The Cahn argument [56] does not apply to the systems considered here, since the bulk correlation length stays finite at any temperature. Therefore, it is possible that under certain conditions a complete wetting not appear at all, independently of the temperature.

In this work we confine the discussion to the systems with $u_{BB}=0$, since this case corresponds more closely to the usually studied models of Janus particles.

**Figure 14:**
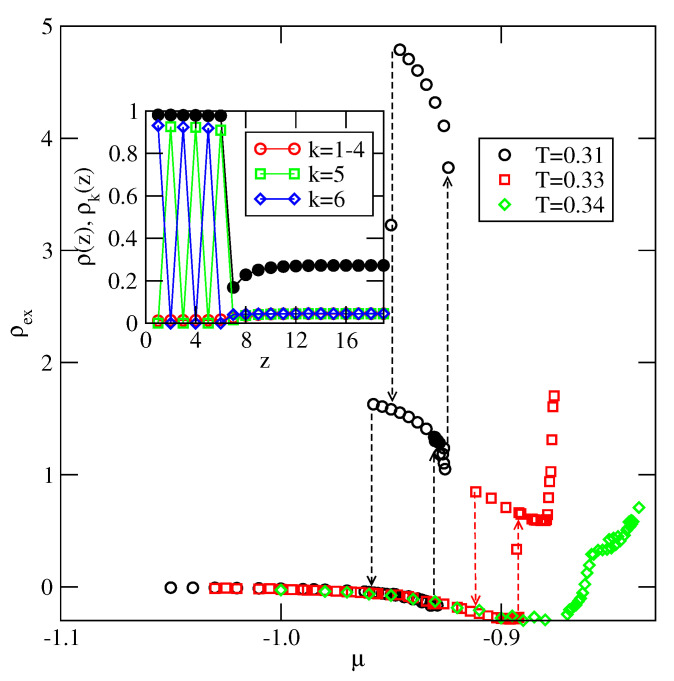
The adsorption isotherms for the system with $u^{*}=0.0$ and $V_{o}=-0.01$ at the temperatures given in the figure. The inset shows the profiles ρ(z), $\rho_{k}\left(z\right)$, recorded at T=0.31 and μ=−0.93.

From the ground state considerations if follows that the systems with $u_{BB}=0$ are expected to exhibit a complete wetting at any temperature down to zero, even for very weakly attractive surfaces. In order to verify this prediction, we have performed calculations for the system characterized by very weak, and nonselective (Δ=0), surface potential, with $V_{o}=-0.01$. At T=0 the layering transitions occur at the chemical potentials very close to the bulk coexistence. In particular, the first layering transition, 0→2, is located at $\mu^{*}=-0.562501E-02$. Finite size effects make it rather difficult to obtain reliable results from simulations carried out for relatively small simulation cells [55]. In particular, at low temperatures, due to strong metastability effects. We have been able to obtain meaningful results at the temperatures above 0.30. Figure 14 shows the adsorption isotherms recorded at T=0.31, 0.33 and 0.34. At low values of chemical potential the surface excess densities are negative, since the attractive interactions in the bulk dominate over the interactions of particles with the surface. Only when μ is close to the bulk coexistence, the surface excess density becomes positive, and multilayer films develop. The isotherm at T=0.31 shows that the layering transitions involve a simultaneous condensation of two layers. At T=0.33 only the first layering transition is preserved, while at T=0.34 the film grows continuously The inset to Figure 14 gives the profiles of local density and local densities of differently oriented particles, recorded at T=0.31 for the 6-layer thick film. These profiles demonstrate that the stacking is 656565, as predicted by the ground state calculations.

**Figure 15:**
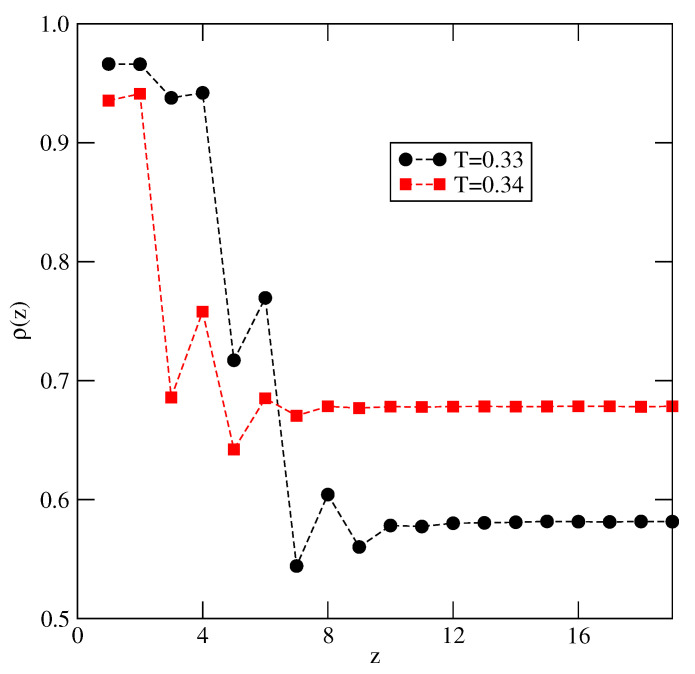
The local density profiles recorded at T=0.33 and μ=−0.88 (circles) and at T=0.34 and μ=−0.84.

At the temperatures equal to 0.33 and 0.34, the adsorbed films also become quite thick when the chemical potential approaches bulk coexistence, though the surface excess densities are rather low, since the dilute phase attains high density. Figure 15 shows the local density profiles recorded at T=0.33 and 0.34, for the chemical potentials close to the bulk coexistence. At T=0.33 the film consists of eight layers, while at T=0.34, the profile shows that only the first four layers have the densities exceeding the bulk density. However, the calculated local density profiles of differently oriented particles have shown that a partial orientational ordering exists in the first 10 (8) layers at T=0.33 (T=0.34).

The above results confirm that the fluid with $u_{BB}=0$ wets even very weakly attractive surfaces.

**Figure 16:**
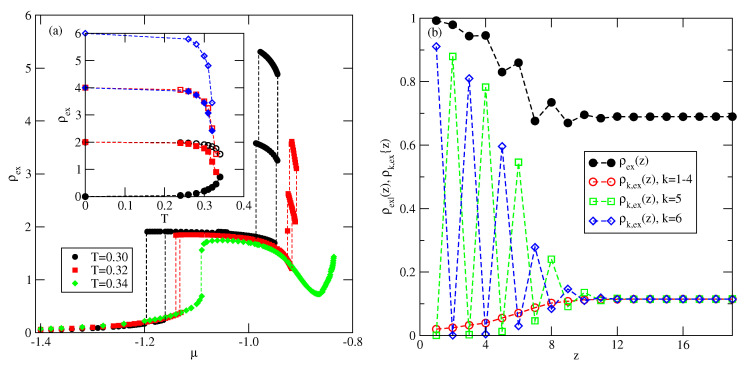
(**a**) The excess adsorption isotherms for the system with $V_{o}=-0.4$. The inset shows the estimated (ρ,T) projection of the phase diagram. (**b**) The profiles ρ(z) and $\rho_{k}\left(z\right)$, recorded at T=0.34 and μ=0.836.

The calculations carried out for several systems with different $V_{o}$, down to −1.0, have demonstrated qualitatively the same behavior. Figure 16a presents explicit results for the system with $V_{o}=-0.4$, which show that the films grow via n→n+2 layering transitions, over a wide range of temperatures. The recorded profiles of $\rho_{k}\left(z\right)$ (see Figure 16b) have also shown that the stacking in multilayer films is the same as in the case of $V_{o}=-0.01$. The inset to Figure 16a shows the estimated part of the (ρ−T) projection of the phase diagram. Our results suggest that the critical temperatures of the layering transition 0→2 is higher than the critical temperatures of subsequent (2→4 and 4→6) layering transitions. Figure 16b gives the local density profiles ρ(z) and $\rho_{k}\left(z\right)$ (k=1,2…,6) recorded at T=0.34 and for the chemical potential close to the bulk coexistence ($\mu^{*}\approx 0.01$). At this temperature, the density of the bulk disordered dilute phase is equal to about 0.7. The profiles demonstrate that the film quite thick, and exhibits the stacking predicted by the ground state considerations.

From the calculations carried out for the systems with different $V_{o}$ we have found that the critical points of subsequent layering transitions, $T_{c}\left(n\to n+2\right)$, are nearly independent of $V_{o}$. In particular, the critical temperature of the first layering transition, $T_{c}\left(0\to 2\right)$, is located close to 0.34, and the critical temperatures of the higher layering transitions are only very slightly lower than 0.34. This suggests that the roughening temperature in those systems is also close to 0.34.

**Figure 17:**
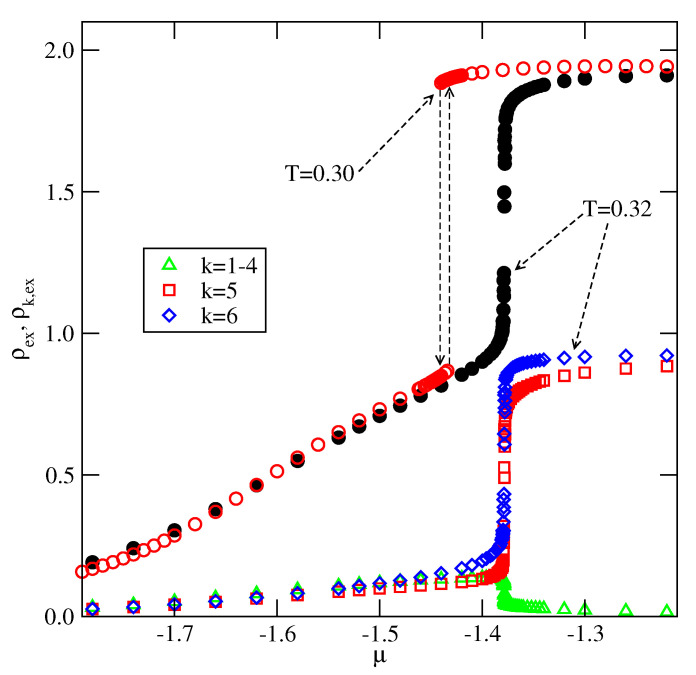
The excess adsorption isotherms for the system with $V_{o}=-1.0$ at T=0.30 and 0.32.

When $V_{o}$ decreases, and approaches the limiting value of −1/0.875, the critical temperature of the first layering transition, 0→2, decreases. For example, for $V_{o}=-1.0$, the recorded isotherms at T=0.30 and 0.32 (see Figure 17) show that the critical temperature of the transition 0→2 is lower than 0.32. It should be also noted that already below the critical temperature of 0.3, the first layer assumes quite high density at the first layering transition point.

**Figure 18:**
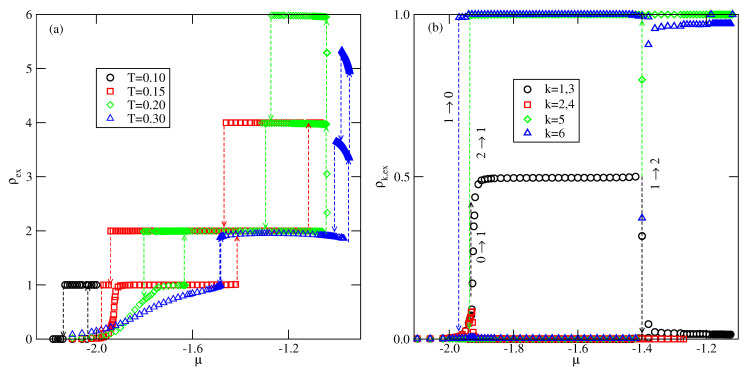
(**a**) The excess adsorption isotherms for the system with $V_{o}=-1.2$. (**b**) The excess densities of differently oriented particles $\rho_{k},ex$, recorded along the adsorption–desorption isotherm at T=0.15.

When $V_{o}$ is lower than −1/0.875 the first layering transition is expected to lead to the monolayer film. We have performed calculations for the system with $V_{o}=-1.2$, and the examples of recorded excess adsorption–desorption isotherms are given in Figure 18.

At sufficiently low temperatures, the first layering transition does lead to the formation of a monolayer film, which is followed by the condensation of the second layer and a series of n→n+2 transitions. At the temperatures up to 0.20, the adsorbing particles in the monolayer predominantly assume in-plane orientations (see Figure 18b). However, when the second layer condenses the particles in the first layer undergo reorientation and the stacking with out-of-plane orientations 6 and 5 develops. On the other hand, when the monolayer film appears due to desorption of particles from the second layer, out-of-plane orientations of particles are preserved. It should be noted that in the case of non-selective substrate, the energy of a monolayer film with the in-plane and out-of-plane orientations of particles is the same. However, the structure of monolayer with in-plane orientations is degenerated, and hence has higher entropy than the structure with out-of-plane orientations. This is the reason why the adsorbing particles prefer in-plane orientations. On the other hand, the bilayer, and thicker films, assume out-of-plane orientations of particles, since such structures are stabilized by the lower potential energy. Upon desorption, the particles in the resulting monolayer do not change their orientations, however. Such a process would require a synchronous reorientation of all particles in the film.

Now we turn to the discussion of results for the systems with selective walls.

**Figure 19:**
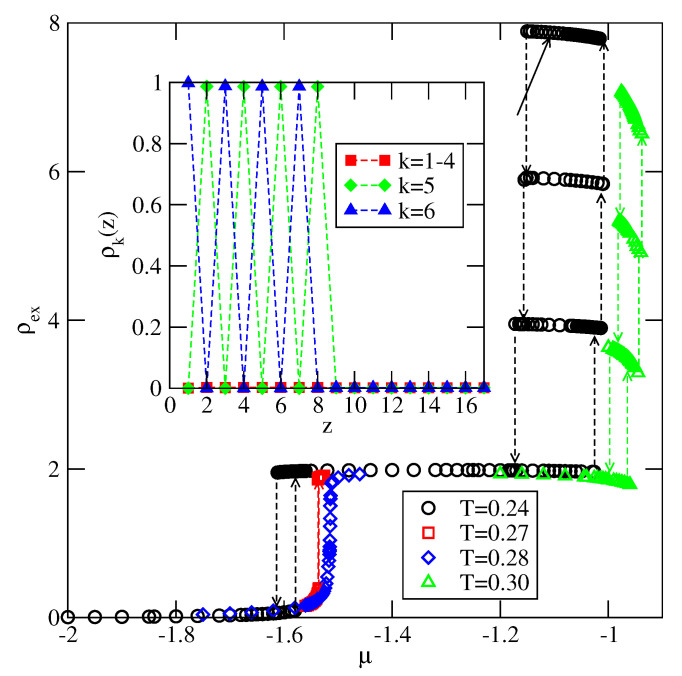
The excess adsorption isotherms for the system with $V_{A}=-0.4$ and $V_{B}=-1.0$. The inset shows the profiles of differently oriented particles, recorded at T=0.24 and μ−1.13 in the film with eight occupied layers.

In the case of $V_{B}<V_{A}$ (ΔV>0), the behavior is qualitatively very similar to the case of non-selective walls. In particular, the particles in the first layer assume the orientation 6, with their B halves oriented to the surface. This stabilizes the stacking with alternate orientations 6565… in thick films. For any $V_{B}\geq -1.0$, the film grows via a series of n→n+2 layering transitions, and only even numbers of occupied layers appear. This scenario is confirmed by the adsorption-desorption isotherms, calculated for several systems. In Figure 19, we present explicit results for $V_{A}=-0.4$ and $V_{B}=-1.0$, and qualitatively the same results have been obtained for other systems with $V_{B}>-1.0$. The main part of Figure 19, presents the adsorption–desorption isotherm at T=0.24, which shows a series of n→n+2 layering transitions. The pronounced hysteresis loops are due to metastability effects. In order to determine the locations of those transitions one would need to calculate free energies of the film consisting of different numbers of filled layers. In this paper, we have concentrated on the qualitative picture only, and hence have not attempted such calculations. The isotherms recorded at T=0.27 and 0.28 show that the critical temperature of the first layering transition, $T_{c}\left(0\to 2\right)$, is located slightly below T=0.28. The calculations of carried out for the system with $V_{A}=-0.4$ and $V_{B}=-0.8$ have shown that $T_{c}\left(0\to 2\right)$ is considerably higher, and equal to about 0.33. Thus, it is only slightly lower than found in the systems with non-selective walls. A decrease of $T_{c}\left(0\to 2\right)$, when $V_{B}$ decreases towards −1.0, may be attributed to a gradual lowering of bilayer stability upon the approach to the regime in which the monolayer becomes stable. This occur when $V_{B}$ becomes lower than −1.0. The inset to Figure 19 shows the local density profiles if differently oriented particles in the film with eight occupied layers, and confirms that the stacking in multilayer films is the same as predicted by the ground state considerations.

**Figure 20:**
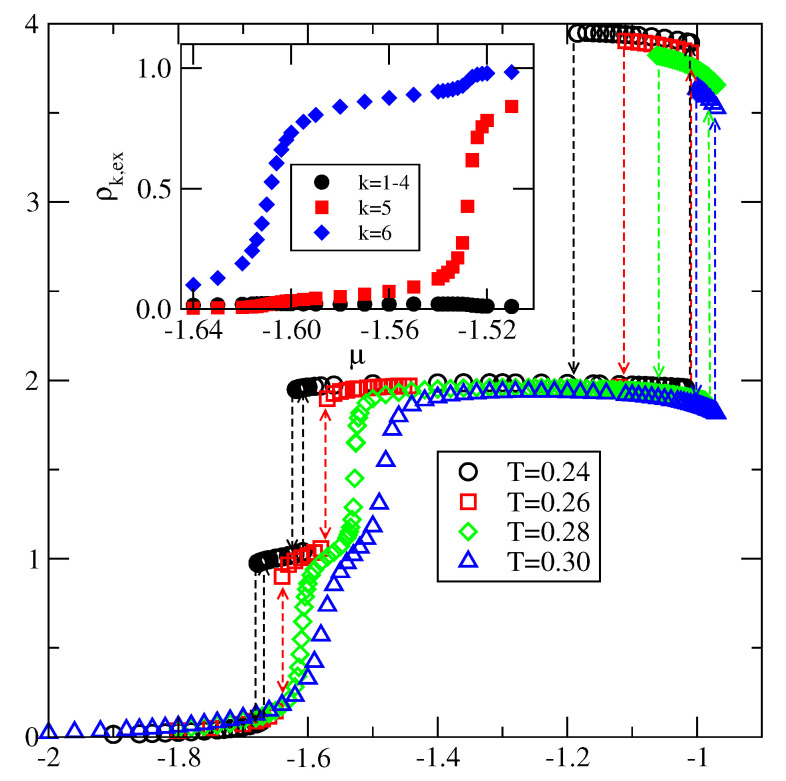
The excess adsorption isotherms for the system with $V_{A}=-0.4$ and $V_{B}=-1.1$. The inset shows the parts of excess isotherms of differently oriented particles at T=0.28 in the region of μ over which the first two layers are formed.

As already mentioned, when $V_{B}<-1.0$ the first layering transition leads to the formation of a monolayer. This transition is followed by the condensation of the second layer. However, a further grow of thicker film occurs, again, via a series of n→n+2 layering transitions. Here, in Figure 20, we present the results for the system with $V_{A}=-0.4$, and $V_{B}=-1.1$. The main figure shows the excess adsorption-desorption isotherms recorded at different temperatures. It is quite well seen that the first layering transition leads to the monolayer film. Then the second layer is formed. It should be noted, that the critical temperatures of these two transitions are practically the same. The isotherm calculated at T=0.27 has been found to already be supercritical, with respect to the both 0→1 and 1→2 transitions. In the inset to Figure 20 we show the excess densities of differently oriented particles along the isotherms at T=0.28. The excess densities of in-plane orientations (k=1,2,3 and 4) remain very low all along the isotherms. In the monolayer the particles predominantly assume the orientation 6, with the B halves of particles directed towards the surface, while the particles in the second layer have opposite orientation, with A halves pointing down. The formation of thicker film involves a simultaneous adsorption in two layers with predominant orientations k=6 and 5, as expected.

All systems with $V_{B}<V_{A}$, exhibit enhanced stability of films with even numbers of filled layers, even when the first layering transition leads to the formation of a monolayer.

In the case $V_{A}<V_{B}$ (ΔV<0), the behavior is different, though the structure of adsorbed films also depend only on $V_{A}$ and $V_{B}$. An important difference between the systems with ΔV lower and higher than zero is that in the former stable monolayer films appear for considerably weaker attraction between the fluid particles and the surface. This has already been demonstrated in Section 3. Here we consider the systems with $V_{B}=-0.4$ and different $V_{A}$, between −0.6 and −1.0.

**Figure 21:**
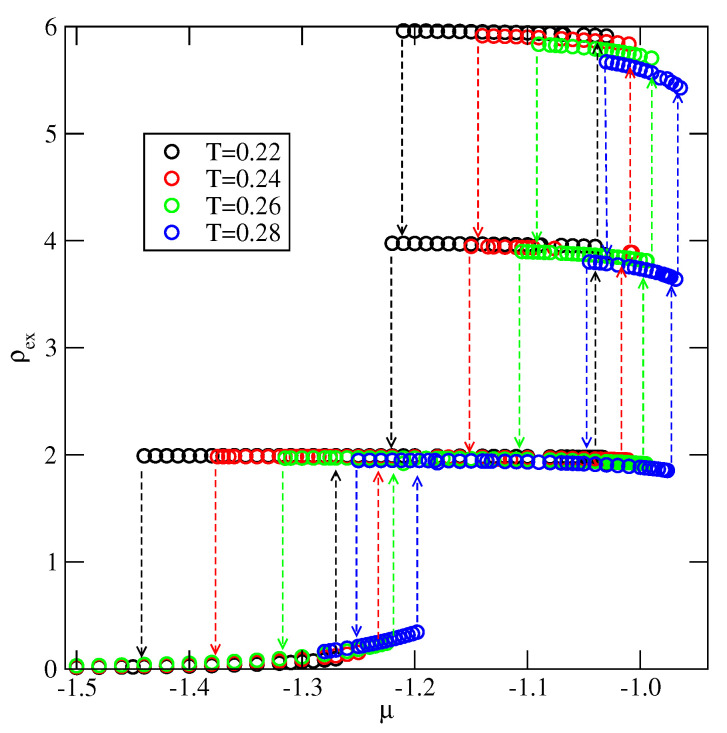
The excess adsorption isotherms for the system with $V_{A}=-0.6$ and $V_{B}=-0.4$.

**Figure 22:**
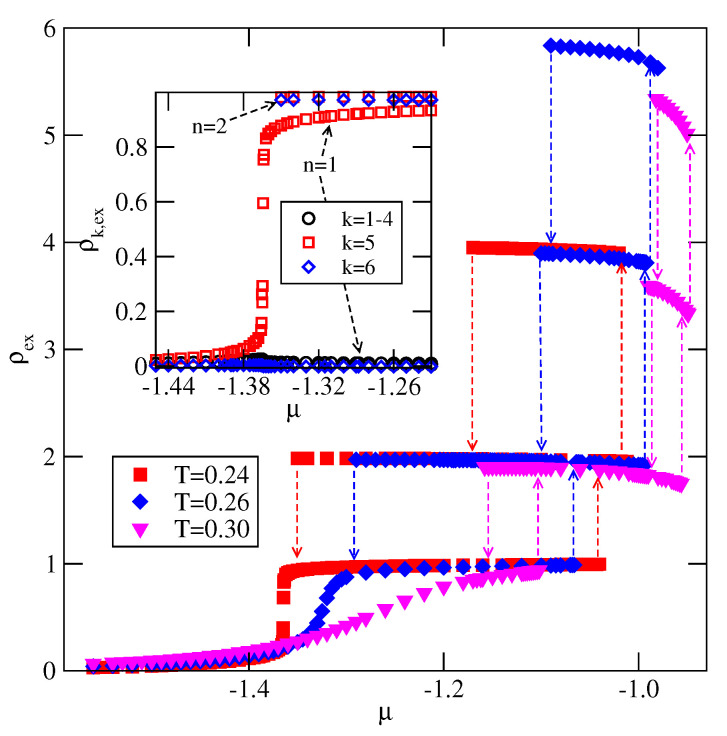
The excess adsorption isotherms for the system with $V_{A}=-0.8$ and $V_{B}=-0.4$. The inset shows changes of excess densities of differently oriented particles along the parts of adsorption and desorption isotherms, at T=0.15, in the region of stability of monolayer and bilayer film.

In the case of $V_{B}=-0.6$, the ground state considerations imply that the film should grow via a series of n→n+2 transitions. Indeed, the results given in Figure 21, are in a full agreement with that prediction. In this system the difference between $V_{A}$ and $V_{B}$ is too small to stabilize a monolayer film. Note that at T=0, the 0→1 transition occurs at $\mu^{*}=-0.3$, while the →2 transition is located at μ**=−0.25. The isotherms given in Figure 22 show that the first layering transition leads to the monolayer, in which the adsorbed particles assume the orientation 5, i.e., with their A halves pointing towards the surface. This transition is followed by the condensation of the second layer. However, the stacking in the bilayer film is 65 rather than 56. A further growth of the film occurs via a series of n→n+2 transitions, and the stacking does not change.

**Figure 23:**
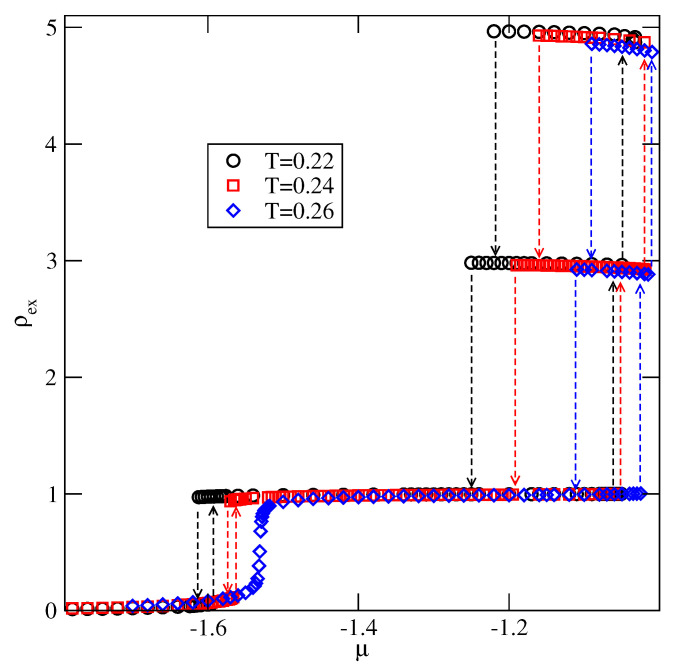
The excess adsorption isotherms for the system with $V_{A}=-1.0$ and $V_{B}=-0.4$.

In the last of considered here systems, with $V_{A}=-1.0$, the film exhibits only odd numbers of filled layers. The examples of adsorption–desorption isotherms are shown in the main part of Figure 23. The inset to Figure 23 gives the excess adsorption–desorption isotherms of differently oriented particles at T=0.24. From the presented results it follows that the developing film has the stacking 5656…. Again, the results agree very well with the predictions stemming from the ground state calculations.

## 6. Final Remarks

In this work, we have discussed the formation of multilayer films by Janus-like particles on non-selective and selective walls using a simple lattice model. The interaction between a pair of particles has been limited to the first nearest neighbors, and assumed to depend on their mutual orientations. In particular, we have assumed that $u_{AA}-1.0$ and $u_{AB}=0$. The ground state calculations have shown that the phase behavior strongly depends on the strengths of surface potential felt by the A and B halves of Janus particles, as well as on the interaction energy between their B halves ($u_{BB}$). It has been shown that in the particular case of $u_{BB}=0$, a complete wetting should occur even for very weakly attractive surfaces. On the other hand, for negative $u_{BB}$ (attractive BB interaction), a complete wetting at T=0 was found to occur only for sufficiently strongly attractive fluid–wall interactions. This agrees with the previous results obtained for the systems with isotropic interactions [12], which demonstrated that the formation of multilayer films and wetting behavior is determined by the relative strengths of fluid-fluid and fluid-wall interactions.

We have estimated bulk phase diagrams for several system characterized by different $u_{BB}$ between 0 and −1.0. It has been shown that all exhibit qualitatively the same behavior. In particular, a high stability of the ordered high density phase (cf. Figure 2) suppresses the existence of a fluid, and hence the phase diagrams have a swan neck shape. It has been also demonstrated than at ρ=1, the bulk phase undergoes a discontinuous (first-order) order order-disorder transition.

Explicit results for nonuniform systems at finite temperatures, also obtained via Monte Carlo simulations in the grand-canonical ensemble, have been presented only for the systems with $u_{BB}=0$. We have confirmed that a complete wetting occurs even for very weakly attractive surface. In general, the formation of multilayer films has been shown to agree very well with the results of ground state calculations. At low temperatures, the systems with $V_{A}=V_{B}$, and with $V_{A}>V_{B}$, develop via a sequence of n→n+2 layering transitions. Only when the surface potential becomes strong enough the first layering transition leads to the monolayer film, but a further grow of the adsorbed layer involves only even numbers of occupied layers.

When $V_{A}<V_{B}$, a sequence of layering transitions and the stacking of multilayer films have been found to depend on $V_{A}$ and on ΔV. For $V_{A}$ larger than about −0.51 the film grows via a sequence of n→n+2 transitions, while for lower values of $V_{A}$, the first layering transition leads to the monolayer film. What happens next depends on ΔV. For |ΔV| smaller than about 0.55, the monolayer undergoes the transition to the bilayer, and a further film development involves n→n+2 transitions. When |ΔV| is larger than about 0.55, the multilayer films consist of only odd numbers of layers.

The formation of multilayer films and. in particular, wetting behavior is expected to change when the BB interaction becomes attractive ($u_{BB}<0$). Already the ground state behavior have shown that when the strength of attractive BB interaction increases, a complete wetting occurs at T=0 only when the fluid-surface interactions ar sufficiently strong. It is expected that in the systems with weaker surface potential, a complete wetting occurs at, and above, a certain wetting temperature, which depends on $u_{BB}$, $V_{A}$ and $V_{B}$. These problems will be considered in our future paper.

## Figures and Tables

**Table 1:** Different possible values of the nearest neighbor interaction energy.

$u_{1}$	=	$u_{AA}$
$u_{2}$	=	$u_{AB}$
$u_{3}$	=	$u_{BB}$
$u_{4}$	=	$0.5\;(u_{AB}+u_{AA})$
$u_{5}$	=	$0.5\;(u_{AB}+u_{BB})$
$u_{6}$	=	$0.5\;(u_{AA}+u_{BB})$
$u_{7}$	=	$0.25\;\left(u_{AA}+u_{BB}\right)+0.5u_{AB}$

**Table 2:** Interlayer energies between neighboring layers (per site) for differently oriented particles. In this table, the entry 1 for $k_{l-1}$ and $k_{l}$ corresponds to any of the in-plane orientation (k=1,2,3 or 4.)

$k_{l-1}$	$k_{l}$	$u_{\mathrm{inter}}\left(k_{l-1},k_{l}\right)$
5	5	0
5	6	$u_{BB}$
6	6	0
6	5	−1.0
5	1	$0.5u_{BB}$
6	1	−0.5
1	5	−0.5
1	6	$0.5u_{BB}$
1	1	$0.5\;(u_{BB}-1.0)$
